# Female patients with low systemic BMD are prone to bone loss in Gruen zone 7 after cementless total hip arthroplasty: A 2-year DXA follow-up of 39 patients

**DOI:** 10.3109/17453670903316801

**Published:** 2009-10-01

**Authors:** Jessica J Alm, Tatu J Mäkinen, Petteri Lankinen, Niko Moritz, Tero Vahlberg, Hannu T Aro

**Affiliations:** ^1^Orthopaedic Research Unit, Department of Orthopaedic Surgery and Traumatology, Turku University Central Hospital and University of TurkuFinland; ^2^Department of Biostatistics, University of TurkuTurkuFinland

## Abstract

**Background and purpose** Factors that lead to periprosthetic bone loss following total hip arthroplasty (THA) may not only depend on biomechanical implant-related factors, but also on various patient-related factors. We investigated the association between early changes in periprosthetic bone mineral density (BMD) and patient-related factors.

**Patients and methods** 39 female patients underwent cementless THA (ABG II) with ceramic-ceramic bearing surfaces. Periprosthetic BMD in the proximal femur was determined with DXA after surgery and at 3, 6, 12, and 24 months. 27 patient-related factors were analyzed for their value in prediction of periprosthetic bone loss.

**Results** Total periprosthetic BMD was temporarily reduced by 3.7% at 3 months (p < 0.001), by 3.8% at 6 months (p < 0.01), and by 2.6% at 12 months (p < 0.01), but recovered thereafter up to 24 months. Preoperative systemic osteopenia and osteoporosis, but not the local BMD of the operated hip, was predictive of bone loss in Gruen zone 7 (p = 0.04), which was the only region with a statistically significant decrease in BMD (23%, p < 0.001) at 24 months. Preoperative serum markers of bone turnover predicted the early temporary changes of periprosthetic BMD. The other patient-related factors failed to show any association with the periprosthetic BMD changes.

**Interpretation** Female patients with low systemic BMD show greater bone loss in Gruen zone 7 after cementless THA than patients with normal BMD. Systemic DXA screening for osteoporosis in postmenopausal patients before THA could be used to identify patients in need of prophylactic anti-resorptive therapy.

## Introduction

Stress-shielding seems to be the most important single factor causing bone loss in the femur after cementless total hip arthroplasty (THA) (Tanzer et al. 2001, Sköldenberg et al. 2006). However, various patient-related factors may also be involved (Venesmaa et al. 2001, Aldinger et al. 2003). Some studies have suggested that the rate of bone turnover determined by metabolic bone markers (Yamaguchi et al. 2003) and systemic bone mineral density (BMD) measured from the lumbar spine, contralateral hip and forearm (Rahmy et al. 2004, Grochola et al. 2008, van der Wal et al. 2008) can predict periprosthetic bone loss. The process is believed to carry a risk of implant failure and difficulty with the performance of a revision surgery (Haddad et al. 1999). Thus, it may be an indication for preventive anti-resorptive therapy (Bhandari et al. 2005).

Undiagnosed osteoporosis is surprisingly common in patients with hip osteoarthritis (OA) (Glowacki et al. 2003). A recent study from our group also showed a high rate of primary and secondary osteoporosis in females with advanced OA of the hip (Mäkinen et al. 2007). These patients have now undergone cementless THA and we have evaluated them prospectively for the influence of various patient-related factors on periprosthetic bone remodeling in the proximal femur.

## Patients and methods

### Inclusion and exclusion criteria

The original patient population consisted of 61 consecutive osteoarthritic women undergoing THA surgery between August 2003 and March 2005. The Ethics Committee of the Hospital District of Southwest Finland approved the study protocol (16.04.2002, # 4/2002§76). Informed consent was obtained from all patients. The exclusion criteria applied were: Paget's disease, disorder of parathyroid function, and/or treatment with corticosteroids, bisphosphonates, or calcitonin. 8 patients were excluded due to previously diagnosed osteoporosis or systemic corticosteroid use. 10 patients were excluded because of severe osteoporosis (T-score < –3.5 at any anatomical location) requiring the initiation of anti-resorptive therapy. Of the 43 patients who fulfilled the study criteria, 2 patients could not complete the study protocol due to surgical complications (periprosthetic fracture) and 2 patients were excluded from the analysis because of omission of baseline DXA measurements. Thus, 39 patients were enrolled (Table 1). The Z-scores showed a high variance. Still, the mean Z-scores were relatively high (1.16–1.33), indicating that the study cohort was representative of OA patients with a trend of having BMD in the upper scale of the normal range (Dequeker et al. 2003).

**Table 1.** Demographic data, preoperative DXA, and radiographic findings

Patient characteristics		DXA and radiography	
Age (years)	63 (41–79)	Z-scores	
Height (cm)	162 (SD 6)	Lumbar spine (L1-L4 total)	1.33 (SD 1.24)
Weight (kg)	81 (SD 17)	Contralateral hip (total)	1.17 (SD 0.92)
BMI	31 (SD 6.3)	OA hip (total)	1.16 (SD 0.98)
Previous fractures (n)	10	Non-dominant forearm	1.19 (SD 0.86)
Postmenopausal (n)	36	Systemic BMD (T-scores)	
S-25(OH)D < 50 nmol/L (n)	15	Normal BMD (n)	12
Smokers (n)	4	Osteopenia (n)	22
Alcohol consumption:		Osteoporosis (n)	5
1–5 drinks/week	13	Kellgren-Lawrence score:	
6–10 drinks/week	7	2 (n)	3
WOMAC score	51 (SD 16)	3 (n)	17
Harris hip score	49 (SD 15)	4 (n)	19
		Canal flare index:	
		Stovepipe (n)	4
		Normal (n)	31
		Champagne-Flute (n)	4
		Dorr classification:	22
		Type A (n)	16
		Type B (n)	1
		Type C (n)	

### Preoperative evaluation

To assess functional disability, the patients were evaluated with the Harris hip score and the Western Ontario and McMaster Universities osteoarthritis index (WOMAC).

Radiographic OA was classified by 2 independent observers using the Kellgren-Lawrence grading system. The shape and the bone quality of the proximal femur were assessed by the qualitative classification into 3 distinct pattern types (Dorr type A, B, or C). The canal flare index (CFI) was calculated from digital radiographs (Noble et al. 1998). Briefly, the metaphyseal width 20 mm proximal to the most prominent point of the lesser trochanter (D) and the intramedullary femoral isthmus width (G) were measured. CFI was calculated as the ratio of D to G, and the canal shapes of the femurs were classified as normal (3-4.7), stovepipe (< 3), or champagne-flute (> 4.7) (Table 1). These parameters were statistically tested as radiographic predictors of periprosthetic bone loss.

Standard laboratory tests were done in order to detect metabolic bone disorders and vitamin D insufficiency, as described previously (Mäkinen et al. 2007). Complete blood cell count, plasma calcium (P-Ca), serum ionized calcium (S-Ca-Ion), plasma phosphorus (P-Pi), serum parathyroid hormone (S-PTH), and 25-hydroxyvitamin D (S-25(OH)D) were measured. Biochemical markers of bone turnover were determined in order to evaluate the rate of bone formation and resorption as predictors of periprosthetic bone loss. 3 biochemical serum markers—osteocalcin (OC), bone alkaline phosphatase (bone ALP), and intact procollagen type I N propeptide (intact PINP)—were used to assess the rate of bone formation. To evaluate the rate of bone resorption, serum levels of C-terminal crosslinking telopeptide of type I collagen (CTX), N-terminal crosslinking telopeptide of type I collagen (NTX), and tartrate-resistant acid phosphatase type 5b (TRACP 5b) were assayed as described previously (Mäkinen et al. 2007).

### DXA measurements

Bone mineral density (BMD) was measured with a Hologic QDR 4500C densitometer (Hologic Inc., Waltham, MA) at the lumbar spine (from L1 to L4), proximal femurs, and distal non-dominant forearm. Based on the DXA results, the patients were divided into 3 groups: normal BMD, osteopenia, or osteoporosis (Table 1). A patient was classified as osteopenic if she had a T-score of between –1 and –2.5 in any of the aforementioned densitometry sites. Correspondingly, a patient with a T-score of less than –2.5 was classified as osteoporotic.

**Figure 1.**
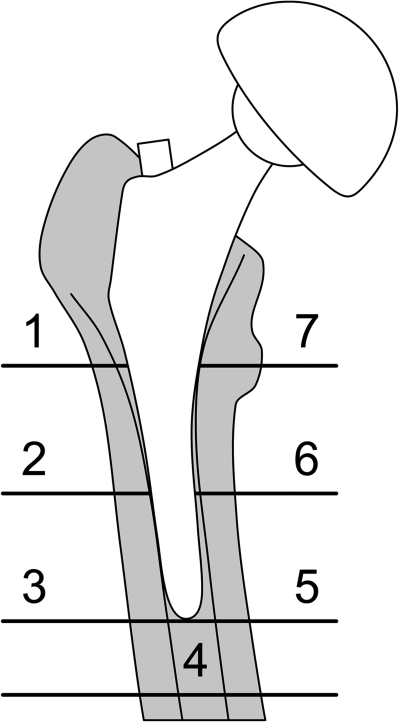
Gruen zones used in DXA analysis of periprosthetic BMD.

DXA measurement of periprosthetic BMD was performed within 7 days of surgery (baseline) and the measurement was repeated at 3, 6, 12, and 24 months. During successive DXA scans, the patient's leg was positioned in a standard neutral rotation using a supporting device. Data were analyzed using software provided by the manufacturer (Metal Removal Option, Hologic). Periprosthetic BMD in the proximal femur was determined from 7 regions of interest (ROIs) (Figure 1) based on Gruen zones. BMD data of zones 1 through 7 were also combined to form a total periprosthetic BMD. The precision error of the Hologic DXA system was measured in 59 double scans performed at different follow-up time points. The precision error varied from 1.5% to 3.4% depending on the ROIs, with an average precision error of 2.3%, which is comparable to other studies (Venesmaa et al. 2001).

### Surgery

The patients underwent cementless THA (Anatomic Benoist Girard II, ABG II, Stryker). The shape of the femoral stem of the prosthesis is meant to follow the anatomical contours of the proximal femur in all 3 dimensions, in order to ensure that the load transfer pattern imitates (as closely as possible) the natural distribution of bone stress within physiological limits (van Rietbergen and Huiskes 2001). The stem is made of titanium alloy with a low modulus (TMZF, 85 GPa) and has a proximal hydroxyapatite (HA) coating. The distal part of the stem has been made short and undersized, and its surface has been ultrapolished to avoid distal bonding. The stem sizes used were 3 (17 patients), 4 (9 patients), and 5 (13 patients). Thus, the average stem size was 4.6. The cementless press-fit cups were also HA-coated. Ceramic heads (28 mm) and ceramic liners (both made of aluminium oxide ceramic, Al_2_O_3_) were used. The patients were operated according to standard techniques using an anterolateral Hardinge approach. According to the prevailing clinical practice, the patients were instructed to perform partial weight bearing; this was followed by full weight bearing after 6 weeks.

### Statistics

The main variable investigated, the time-related change in BMD in the periprosthetic regions, was analyzed using analysis of variance for repeated measurements with Bonferroni's correction for multiple comparisons between the time points.

The statistical significance of preoperative patient-related factors for prediction of time-related periprosthetic BMD changes was evaluated using analysis of variance for repeated measurements (categorical factors) and analysis of covariance for repeated measurements (continuous factors) with Tukey's adjustment for multiple comparisons. The significance of each patient-related factor for the observed changes in BMD at the endpoint was determined using linear models with calculation of the coefficients of determination (R^2^) for these models. Continuous variables were treated as covariates, while categorical variables were treated as fixed factors with Tukey's adjustment for multiple comparisons when comparing the categories. The demographic patient-related factors investigated included age, body mass index, serum vitamin D level, previous fractures, alcohol consumption, and preoperative WOMAC and Harris hip scores. Also, the radiological parameters CFI, Dorr classification and Kellgren-Lawrence scores were analyzed for their value as predictors of periprosthetic BMD changes. When investigating the association between the rate of bone turnover and changes in periprosthetic BMD, biochemical serum markers were analyzed by quartiles with the two middle quartiles combined (lowest 25%, middle 50%, and highest 25%). In addition, the uncoupling index was calculated in order to evaluate the balance of bone turnover, taking into account all 6 markers of bone formation and resorption (Eastell et al. 1993).

The association between preoperative BMD and periprosthetic bone loss in the femur was investigated using both the local BMD of the operated hip and the systemic BMD (based on the lowest T-scores).

Differences in absolute BMD of Gruen zone 7 between the 3 patient groups (normal BMD, osteopenia, osteoporosis) were analyzed at baseline and at 24 months using one-way analyses of variance with Tukey's adjustment for multiple comparisons.

For Gruen zone 7, the association between baseline BMD and absolute loss in BMD (g/cm^2^) as well as the association between change in BMD (%) and the lowest preoperative systemic T-score were analyzed with linear regression and presented with the coefficient of determination (R^2^).

Statistical analyses were done using SAS software for Windows, release 9.1 (SAS Institute Inc., Cary, NC). Linear regression was done using SPSS version 16.0 for Windows. P-values less than 0.05 were considered statistically significant.

## Results

### Clinical outcome

All 39 patients completed the study protocol with sequential clinical and radiographic evaluation and periprosthetic DXA measurements up to 24 months. None of them showed radiographic signs of component loosening or periprosthetic osteolysis. The functional outcome of the THA evaluated with Harris hip score improved from an average preoperative score of 49 (13–75) points to 84 (47–100) points at 24 months. The WOMAC score also improved from 51 (33–95) points to 15 (0–59) after surgery.

### Periprosthetic BMD changes

**Figure 2.**
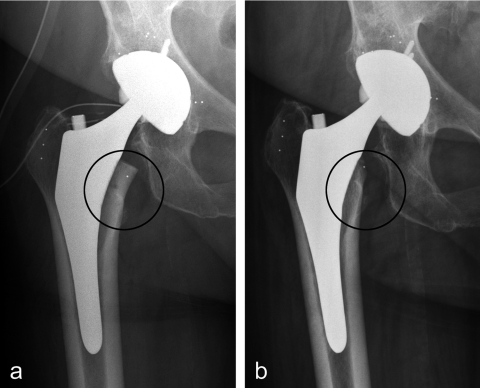
An ABG II hip prosthesis postoperatively (a) and at 24 months (b). Bone loss in Gruen zone 7 was seen as rounding off of the medial femoral neck (shown with circle).

Gruen zones 1, 2, and 3 showed transient decrease in BMD during the first 6 months after THA but recovered thereafter (Table 2). Total periprosthetic BMD became temporarily reduced by 3.7% at 3 months (p < 0.001), by 3.8% at 6 months (p < 0.01), and by 2.6% at 12 months (p < 0.01), but it approached the baseline value by 24 months (Table 2). At 24 months, 2 zones showed a statistically significant change in BMD compared to baseline: Gruen zone 5 with a 5.2% increase (p < 0.001) and Gruen zone 7 with a 23% decrease (p < 0.001). Radiographs showed cortical bone rounding in Gruen zone 7 (Figure 2) as a sign of adaptive remodeling.

**Table 2.** Mean BMD of periprosthetic bone and percentage change during follow-up, with 95% confidence intervals

Gruen	BMD (g/cm^2)^					Percentage change from baseline			
zones	Baseline	3 months	6 months	12 months	24 months	3 months	6 months	12 months	24 months
1 mean	0.71	0.68	0.68	0.67	0.67	–2.5 **^a^**	–2.9	–3.7	–3.1
95% CI	0.66–0.75	0.64–0.72	0.64–0.72	0.63–0.71	0.63–0.71	–5.6 to 0.67	–7.4 to 1.7	–9.2 to 1.9	–8.4 to 2.3
2 mean	1.29	1.22	1.22	1.24	1.25	–5.5 **^b^**	–5.8 **^c^**	–3.5	–2.6
95% CI	1.24–1.35	1.16–1.28	1.16–1.27	1.19–1.29	1.20–1.30	–8.3 to –2.6	–8.2 to –3.3	–6.4 to –0.6	–6.0 to 0.8
3 mean	1.53	1.48	1.48	1.50	1.53	–3.5 **^a^**	–3.0 **^a^**	–1.9	0.7
95% CI	1.49–1.57	1.42–1.53	1.43–1.53	1.45–1.55	1.48–1.59	–5.9 to –1.1	–5.1 to –0.9	–4.1 to 0.4	–1.9 to 3.3
4 mean	1.67	1.65	1.65	1.67	1.69	–1.5	–1.1	0.1	1.1
95% CI	1.62–1.73	1.58–1.71	1.60–1.71	1.62–1.73	1.63–1.75	–2.9 to –0.2	–2.3 to 0.1	–0.9 to 3.0	–1.5 to 1.6
5 mean	1.55	1.56	1.59	1.61	1.63	0.8	2.2 **^a^**	3.5 **^c^**	5.2 **^c^**
95% CI	1.50–1.60	1.51–1.62	1.54–1.63	1.56–1.65	1.59–1.68	–1.2 to 2.7	0.74–3.7	1.9 –5.0	3.1–7.3
6 mean	1.31	1.27	1.26	1.29	1.31	–3.1	–3.5	–1.3	–0.7
95% CI	1.24–1.37	1.20–1.33	1.19–1.32	1.22–1.36	1.24–1.38	–5.7 to –0.4	–6.5 to –0.6	–4.6 to 1.9	–4.2 to 2.8
7 mean	1.12	0.93	0.88	0.88	0.87	–16 **^c^**	–21 **^c^**	–21 **^c^**	–23 **^b^**
95% CI	1.04–1.19	0.86–1.0	0.80–0.96	0.80–0.96	0.79–0.96	–20 to –13	–25 to –17	–26 to –17	–28 to –18
Total BMD	1.23	1.18	1.18	1.19	1.21	–3.7 **^c^**	–3.8 **^b^**	–2.6 **^b^**	–1.5
95% CI	1.19–1.27	1.14–1.22	1.14–1.22	1.16–1.23	1.17–1.25	–5.2 to –2.2	–5.2 to –2.5	–4.3 to –1.0	–3.4 to 0.4
**^a^** p<0.05, **^b^** p<0.01, **^c^** p<0.001, p-values with Bonferroni corrections: significant changes in BMD compared to baseline values.									

### Patient-related factors in periprosthetic bone remodeling

**Figure 3.**
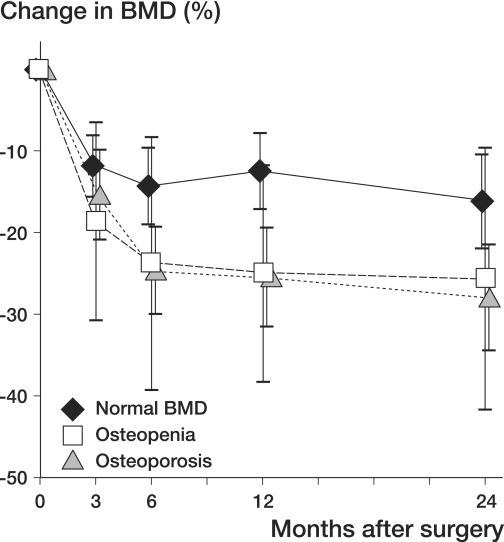
Change in BMD in Gruen zone 7 in THA patients with normal or low systemic BMD, as a function of healing time. In all groups, bone loss in this zone was statistically significant at 24 months (p < 0.001). Osteopenic (n = 22) and osteoporotic (n = 5) patients showed greater bone loss than patients with normal BMD (n = 12) (p = 0.037). Mean values and 95% CIs are given.

**Figure 4.**
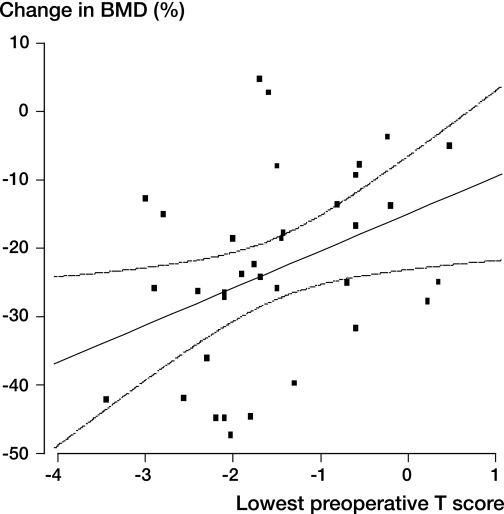
Linear regression (with 95% confidence intervals) demonstrating the association (R^2^ = 0.147, R = 0.38, p = 0.015) between preoperative systemic BMD (lowest preoperative T-score) and change in periprosthetic femur BMD (in Gruen zone 7) at 24 months after THA (n = 36).

The preoperative systemic BMD, evaluated from 3 anatomical locations (contralateral proximal femur, lumbar spine, and non-dominant forearm), predicted bone loss in Gruen zone 7 (p = 0.04). Patients with osteopenia or osteoporosis showed a greater bone loss in Gruen zone 7 than patients with normal systemic BMD (Figure 3). At 24 months, patients with normal systemic BMD showed higher BMD in Gruen zone 7 than osteopenic and osteoporotic patients (p = 0.006 and p = 0.01, respectively). Regression analysis (Figure 4) confirmed that low preoperative systemic BMD was associated with higher bone loss in Gruen zone 7 (R^2^ = 0.15, R = 0.38, p = 0.02). The local BMD (preoperative total BMD of the operated femur) did not act as an independent predictor of periprosthetic bone loss in Gruen zone 7. In Gruen zone 7, no association was found between the baseline BMD and loss of BMD at 24 months.

High preoperative levels of the resorption marker NTX were predictive of bone loss at 3 and 6 months (p < 0.001 and p = 0.001, respectively) in Gruen zone 2. TRACP-5b, another resorption marker, was also associated with bone loss in the same zone at 3 months (p < 0.001) and a low level of bone formation marker intact PINP was predictive of the transient bone loss in Gruen zone 2 (p = 0.01) at 6 months. Uncoupling index also predicted changes in BMD in Gruen zone 2 (p = 0.002). Patients with positive index (turnover in favor of bone formation) showed better preservation of periprosthetic bone than those with negative index (turnover in favor of bone resorption). High preoperative serum bone ALP predicted the continuous increase in BMD (p = 0.04) in Gruen zone 5.

None of the radiographic parameters of the proximal femur morphology (Table 1) predicted periprosthetic bone loss. The percentage decrease in the total periprosthetic BMD at 3 and 6 months was greater in patients with baseline alcohol intake of 6–10 drinks per week than in non-users (p = 0.01), but at 12 and 24 months there were no differences in the absolute BMD values between alcohol users and non-users.

## Discussion

Unexpectedly, this study showed a statistically significant decrease in total periprosthetic BMD only during the first 12 months. At 24 months, only Gruen zone 7 showed statistically significant bone loss. Low systemic BMD (osteopenia or osteoporosis) predicted this local adverse remodeling process. Interestingly, both the local preoperative BMD of the operated hip and the baseline BMD of Gruen zone 7 failed to act as independent predictors of the process, most likely due to the secondary changes in BMD in the femoral neck and trochanteric region caused by the underlying OA of the hip (Mäkinen et al. 2007). Probably due to the same process, fragility fracture of an OA hip is extremely rare and DXA of the affected hip may give falsely high BMD values for evaluation of systemic osteoporosis, resembling artefacts caused by lumbar spine degeneration.

Total periprosthetic BMD of the proximal femur has been shown to decrease by 5–10% within 2 years after cementless THA (Tanzer et al. 2001, Venesmaa et al. 2001, Kärrholm et al. 2002, Aldinger et al. 2003, Rahmy et al. 2004, Grant et al. 2005, Sköldenberg et al. 2006, van der Wal et al. 2008) with a slow progressive loss or minimal recovery during the following years (Aldinger et al. 2003). Our study demonstrated only temporary loss of total periprosthetic BMD during the first 12 months, followed by recovery thereafter. The maintenance of the periprosthetic bone probably reflects the mechanical characteristics of the femoral stem, confirming the idea that implant-related factors are most critical for preservation of bone stock (van Rietbergen and Huiskes 2001). Previous studies using the same prosthesis have shown a 4.1% decrease in total periprosthetic BMD at 24 months (van der Wal et al. 2006). Several factors could explain our favorable results. The averaged stem size was somewhat larger than in previous studies (van der Wal et al. 2006, 2008). A larger stem size in itself can increase periprosthetic bone loss (Sköldenberg et al. 2006) but, on the other hand, stability can be improved by larger stems, thus eliminating distal bone loss seen with smaller ABG II stems (van der Wal et al. 2006). A larger stem may also help to avoid malalignment of the stem, which appears to cause periprosthetic bone loss in ABG II arthroplasties (Panisello et al. 2006).

The major decrease in periprosthetic BMD in Gruen zone 7 is a common finding in cementless THAs (Tanzer et al. 2001, Venesmaa et al. 2001, Aldinger et al. 2003, Yamaguchi et al. 2003, Rahmy et al. 2004, Sköldenberg et al. 2006, van der Wal et al. 2008). The periprosthetic changes in BMD for the cemented stems follow the pattern observed in cementless THAs, with the highest bone loss in Gruen zone 7 (Li et al. 2007). In patients with cemented THAs, low systemic BMD and high bone loss in Gruen zones 7 and 1 have been found to be predictive of late stem loosening (Nixon et al. 2007), but no association between periprosthetic bone loss and stem migration has been shown, as evaluated by RSA (Li et al. 2007). Regarding cementless THA, the long-term effect of periprosthetic bone loss is unclear. This might be a concern, as the contemporary cementless THAs are expected to survive for 30–40 years in middle-aged patients and an increasing number of cementless THAs are being performed in osteoporotic postmenopausal women with an increased life expectancy. Thus, every effort should be made to minimize the loss of periprosthetic bone stock. In a sheep model of cemented hemiarthroplasty, intravenous administration of long-lasting zoledronic acid was found to reduce cortical osteopenia in Gruen zone 7 (Goodship et al. 2008). Also, several human studies have indicated the efficacy of other bisphosphonates in prevention of periprosthetic bone loss (Wilkinson et al. 2001, Bhandari et al. 2005). Extending previous observations (Rahmy et al. 2004, van der Wal et al. 2008, Grochola et al. 2008), the current study demonstrated higher periprosthetic bone loss in Gruen zone 7 in patients with low systemic BMD. These patients may represent an ideal target group of THA patients for definitive clinical trials of prophylactic anti-resorptive therapy.

In previous studies, determination of metabolic bone markers has given different results in terms of their reliability for monitoring periprosthetic bone loss (Wilkinson et al. 2001, Yamaguchi et al. 2003, Habermann et al. 2007). In our study, 4 markers and the uncoupling index were able to detect the rapid transient BMD changes in 2 Gruen zones during the first 3-6 months. Such a high turnover of periprosthetic bone represents the healing process of cementless THAs, which is known to mimic fracture healing.

The strength of our study was characterized by 4 factors: (1) the inclusion of a homogenous patient population of the same sex and with well-defined exclusion criteria, (2) the use of an anatomically shaped femoral stem with expected minor stress-shielding effects on the proximal femur, (3) the use of ceramic-ceramic weight-bearing surfaces, and (4) the statistical assessment of many potential patient-related factors. The use of ceramic-ceramic bearing surfaces was expected to minimize generation of wear particles as a potential cause of periprosthetic bone resorption, although a recent study showed no difference in periprosthetic bone loss in patients with ceramic-ceramic articulations as opposed to ceramic-on-polyethylene articulations (Kim et al. 2007). Our study did not address the possible impact of sex, type of cementless stem, or cement fixation on the degree of periprosthetic bone loss. There have been reports using quantitative computed tomography (CT) to measure BMD around prosthetic components (Mueller et al. 2007). The CT technique allows a true 3-dimensional densitometry with high precision. Compared to the conventional DXA used in this study, quantitative CT would certainly bring new insights into the remodeling processes.
